# Control of poorly immunogenic tumors with systemic STING agonist‐loaded liposomes targeting cross‐presenting dendritic cells

**DOI:** 10.1002/cti2.70110

**Published:** 2026-06-01

**Authors:** Bijun Zeng, Meghna Talekar, Thais Aragao‐Horoiwa, Amy Cameron, Guangzu Zhao, Yulia Zybina, Wendy Blumenschein, Karyn van de Mark, Wittaya Suwakulsiri, Gretchen A Baltus, Riccardo Dolcetti, Ranjeny Thomas

**Affiliations:** ^1^ Frazer Institute The University of Queensland Brisbane QLD Australia; ^2^ Merck & Co., Inc. Rahway NJ USA; ^3^ Present address: Peter MacCallum Cancer Centre and University of Melbourne Melbourne VIC Australia; ^4^ Present address: James Cook University Brisbane QLD Australia; ^5^ Present address: CSL Ltd Melbourne VIC Australia

**Keywords:** cross presentation, dendritic cells, liposome, melanoma, STING

## Abstract

**Objectives:**

Cancer vaccines combine tumor antigen and adjuvant to drive an anti‐tumor immune response. Clec9A‐targeted nanoparticles directing CD4 and CD8 neo‐epitopes to cross‐presenting dendritic cells (DCs) stimulate adjuvant‐free therapeutically effective tumor‐specific immunity. However, personalised neo‐epitopes limit the scalability of Clec9A nanoparticles for clinical translation. Since Clec9A^+^ DCs constitutively present relevant neoepitopes in tumors but are suppressed in tumor microenvironments, we developed Clec9A‐targeted STING or RIG‐I liposomes to enhance endogenous tumor immunity.

**Methods:**

Liposomes encapsulating RIG‐I or the STING agonist MSA‐1 and functionalised with Clec9A binding peptide WH were optimised for uptake by Clec9A^+^ conventional and plasmacytoid DCs *in vivo*. Biodistribution after i.v. administration was determined with *in vivo* imaging and cellular uptake and activation with flow cytometry. Plasma was analysed for type 1 interferon (IFN) by ELISA and liver, spleen, tumor, blood, kidney, heart and tumor draining lymph node transcriptomes were analysed with Nanostring. Effects on tumor growth and survival, and tumor‐specific T cells were evaluated using the B16 melanoma model.

**Results:**

Clec9A‐targeting liposomes administered i.v. were distributed to spleen, liver and tumor and were taken up by cross‐presenting and plasmacytoid DCs. Type 1 IFN production and DC, B‐ and T‐cell activation were more effective with i.v. Clec9A‐STING than Clec9A‐RIG‐I liposomes. Intravenous Clec9A‐STING liposomes stimulated IFN‐dependent transcription in blood, lung, tumor, and draining lymph nodes. Intravenous Clec9A‐STING liposomes, or intra‐tumoral Clec9A‐RIG‐I liposomes significantly suppressed B16‐F10 melanoma growth and induced neo‐epitope and survivin‐specific CD4^+^ and CD8^+^ T‐cell responses.

**Conclusions:**

Clec9A‐STING liposomes are a scalable, translatable tumor‐targeted immunotherapy platform.

## Graphical abstract

WH‐peptide‐functionalised liposomes encapsulating STING or RIG‐I agonists target intra‐tumoral Clec9A^+^ cDC1 and pDC, triggering release of type 1 IFN, presentation of CD4 and CD8 endogenous tumor epitopes, expansion of CTL with help from CD4 T cells in spleen, and tumor cell killing.

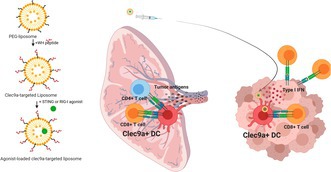

## Introduction

Type I interferons (IFN), particularly IFN‐α, play a critical role in promoting immunogenic presentation of antigens and initiating effective anti‐tumor responses.^1^, ^2^ In dendritic cells (DCs), cytosolic double‐stranded (ds)DNA is sensed by cyclic GMP‐AMP synthase (cGAS), which catalyses the production of the second messenger cGAMP. This, in turn, activates the adaptor protein stimulator of interferon genes (STING), triggering robust type I IFN production and downstream induction of IFN‐stimulated genes (ISG).^3^ Given its capacity to enhance innate and adaptive immunity, STING has emerged as a promising target for cancer immunotherapy. STING is a signalling protein located on the endoplasmic reticulum (ER) membrane. It links the detection of cytosolic DNA to the activation of innate immune response. Upon binding to cGAMP, STING undergoes a conformational change and translocates to the Golgi apparatus, where it activates TANK binding kinase 1 (TBK1).^4^, ^5^ TBK1 phosphorylates interferon regulatory factor 3 (IRF3), which then translocates into the nucleus to drive IFN expression. In DCs, STING signalling enhances antigen processing and cross‐presentation, thereby potentiating CD8^+^ T‐cell responses.^6^ In the tumor microenvironment, activation of this pathway amplifies local inflammatory signals and immune cell recruitment, promoting anti‐tumor immunity. However, despite encouraging results in preclinical mouse models, clinical translation has been limited by challenges such as poor tumor‐specific delivery and systemic toxicity.^7^, ^8^ For example, differences between murine and human STING signalling, including substantial human allelic polymorphism such as the R232, H232 and HAQ variants, and susceptibility to degradation by human phosphodiesterases, significantly impact ligand responsiveness.^9^, ^10^, ^11^ To overcome these limitations, various STING agonists, including cGAMP, have been formulated into a variety of nanoparticles, resulting in protection from enzymatic degradation, enhanced tumor targeting and have improved therapeutic efficacy and survival outcomes in murine models of cancer. These formulations are often complex—posing challenges in standardisation and scalability, potentially impacting the speed of clinical translation. In this respect, the exploitation of cationic liposomes administered intra‐tumorally (i.t.) or intra‐venously (i.v.) may offer a rapid clinical applicability because of their established use and simpler design.^12^, ^13^

MSA‐1, a non‐nucleotide STING agonist which, similar to its analogue MSA‐2, functions as a “molecular glue” to induce STING dimerisation. Unlike traditional cyclic dinucleotides (CDNs) that are susceptible to degradation by human phosphodiesterases, these non‐nucleotide agonists offer enhanced stability and broader potency across diverse human STING molecular variants. Both MSA‐1 and MSA‐2 are water soluble and orally available.^14^, ^15^ Potency of MSA‐2 for STING activation and type 1 IFN production increases upon extracellular acidification, as occurs in the tumor microenvironment (TME). Subcutaneous (s.c.) MSA‐2 administered from the time of tumor implantation reduced tumor volume and/or increased survival in several anti‐PD1‐non‐responsive tumor models, including B16F10, when combined with anti‐PD1.^15^ On the other hand, MSA‐1 required intra‐tumoral (i.t.) administration and when combined with anti‐PD1 was efficacious across non‐responsive tumor models. Of interest, MSA‐1 induced a similar signature of pro‐inflammatory gene upregulation in the tumor by Day 1 to that induced by anti‐PD1 on Day 5. These included DC costimulatory genes *Cd80* and *Cd86* and functional T‐cell activation genes, such as *Gzmb*, *Pfn* and *Ifng*.^14^

Intracellular 5′‐triphosphate RNA with complementary and hairpin loop structures activate type 1 IFN through RIG‐I.^16^ Harnessing their potential in cancer, lipid particle aggregates (LPAs) generated with tumor mRNA induced a type 1 IFN response and DC activation after i.v. administration. When combined with adjuvant cytomegalovirus pp65 epitope‐specific mRNA, these LPAs remodelled glioblastoma TME towards immunogenicity and expanded and activated pp65‐specific CD8^+^ T cells in glioblastoma patients.^17^

Cross‐presenting classical type 1 DCs (cDC1) express cDC1‐specific C‐type lectin receptor Clec9A, which binds its ligand F‐actin, to take up dying or pyroptotic cell antigens and to shuttle F‐actin‐bound proteins into the cross‐presentation pathway.^18^, ^19^ Enhanced antigen cross‐presentation through cDC1 and consequent immunity post‐vaccination has been demonstrated by targeting Clec9A with antigen conjugated with anti‐Clec9A antibodies.^20^, ^21^, ^22^, ^23^ In proof‐of‐concept studies, we demonstrated the pre‐clinical efficacy and mechanism of Clec9A‐targeted tailored nano emulsions (TNE) encapsulating protein antigen or CD4 and CD8 epitopes to stimulate therapeutically‐effective tumor‐specific immunity.^24^ We showed that a single administration of antigen‐Clec9A‐TNE efficiently activated cross‐presenting cDC1 and plasmacytoid DCs, induced secretion of IFN‐α and induced tumor‐specific CTL *in vivo* without additional adjuvant. CTL expansion depended on presentation of CD4 and CD8 cognate epitopes, the presence of CD4 T cells and expression of CD40, MyD88 and IFNAR. After TNE administration, the burst of IFN‐α provoked systemic activation of CD8^+^ and CD8^−^ DCs.^24^ Gou et al. further developed this concept, combining nano‐particulate STING agonist, 2′3′‐cGAMP, with engineered Clec‐9A targeting peptide‐expressing cancer cell membranes. This system induced anti‐tumor effects in anti‐PD‐1‐responsive and unresponsive tumor models.^25^ However, including personalised tumor antigens increases the complexity of nanoparticle production, increasing the time to patient administration. We hypothesised that STING or RIG‐I agonists that stimulate IFN type 1 *in vivo* would increase the immunogenicity of constitutive neo‐epitope presentation by Clec9A^+^ DCs in tumors and tumor draining lymph nodes (dLN) to improve adaptive immunity‐mediated tumor control. As Clec9A^+^ cDC1 infiltrate the immunosuppressive micro‐environment of primary and secondary tumors, we developed a scalable liposomal platform to target cDC1 with STING or RIG‐I agonists administered i.v. or i.t. to activate their capacity to cross‐present naturally processed tumor epitopes in an immunogenic fashion and enhance susceptibility to CD4^+^ and CD8^+^ T‐cell‐mediated tumor control.

## Results

### Designing a scalable, translatable Clec9A‐targeted liposomal system for cancer immunotherapy

**Figure 1:**
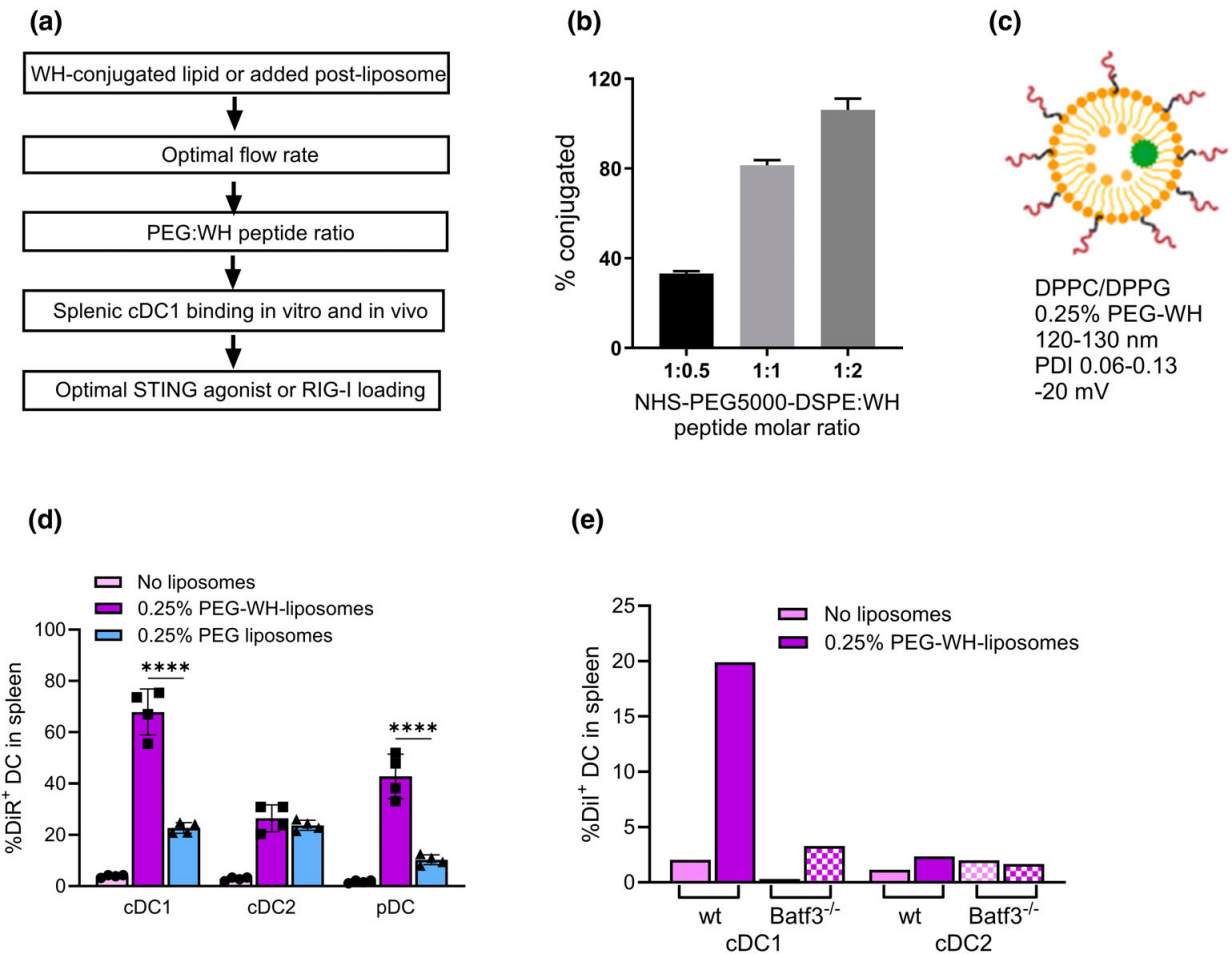
Clec9A‐targeted liposome development. **(a)** Design of experiment strategy for liposome development. **(b)** %WH conjugation with differing PEG:WH peptide molar ratios. **(c)** Schematic representation of the WH‐liposome formulation showing the lipid bilayer (yellow), the WH‐targeting peptide (red) conjugated via PEG‐DSPE spacers, and the encapsulated STING agonist payload (green dot). **(d)** DiR‐labelled non‐targeting liposomes, WH‐liposomes, or no liposomes were administered i.v. and splenic DC uptake assessed 16 h later in C57BL/6 mice. **(e)** DiI‐labelled liposome uptake in Batf3^−/−^ and wild‐type (wt) C57BL/6 mice. All figures are representative of 2–4 replicate experiments. Bars represent mean + SD. ****P < 0.0001.

Liposomes represent a translatable strategy, with their strong clinical track record, versatility, and ease of scale‐up to GMP manufacture for clinical trials. We used a microfluidic system to develop liposomes functionalised with the WH peptide (WH), which has been shown to selectively target mouse Clec9A with no evidence of adverse immune effects,^24^ and loaded with STING or RIG‐I agonists. Figure 1a shows the strategy we adopted to prepare drug loaded non‐targeted and targeted liposomes. During the liposome development, we determined that WH could be conjugated to PEG5000‐DSPE liposomes after liposome production and that conjugation was optimal at a PEG5000‐DSPE:WH molar ratio of 1:2 (Figure 1b). A flow rate of 12 mL/min yielded liposomes with optimal polydispersity index (PDI), intensity, size and charge, with WH‐PEG between 0 and 0.5% acceptable (Supplementary table 1). Characteristics of the final WH‐liposome composition are shown in Figure 1c. Based on the binding of WH‐conjugated liposomes to splenic CD8^+^CD11b^−^ cDC1 relative to CD8^−^CD11b^+^ cDC2 *in vitro*, optimal WH‐PEG was 0.25% (Supplementary table 2). WH‐PEG 0.25% liposomes bound splenic cDC1 and pDC *in vivo* at similarly high levels to those previously reported for WH‐TNEs reference nanoparticle^24^ (Figure 1d). Quantitative differences between *in vitro* and *in vivo* uptake experiments likely reflect physiological factors such as tissue distribution, nanoparticle clearance and the complex microenvironment affecting cellular accessibility *in vivo*. Confirming their specificity, WH‐liposomes were not taken up by splenic DCs in *Batf3* ^*−/−*^ mice that lack cDC1 (Figure 1e, gating strategies in Supplementary figure 1).

### Immune stimulation by STING and RIG‐I liposomes

**Figure 2:**
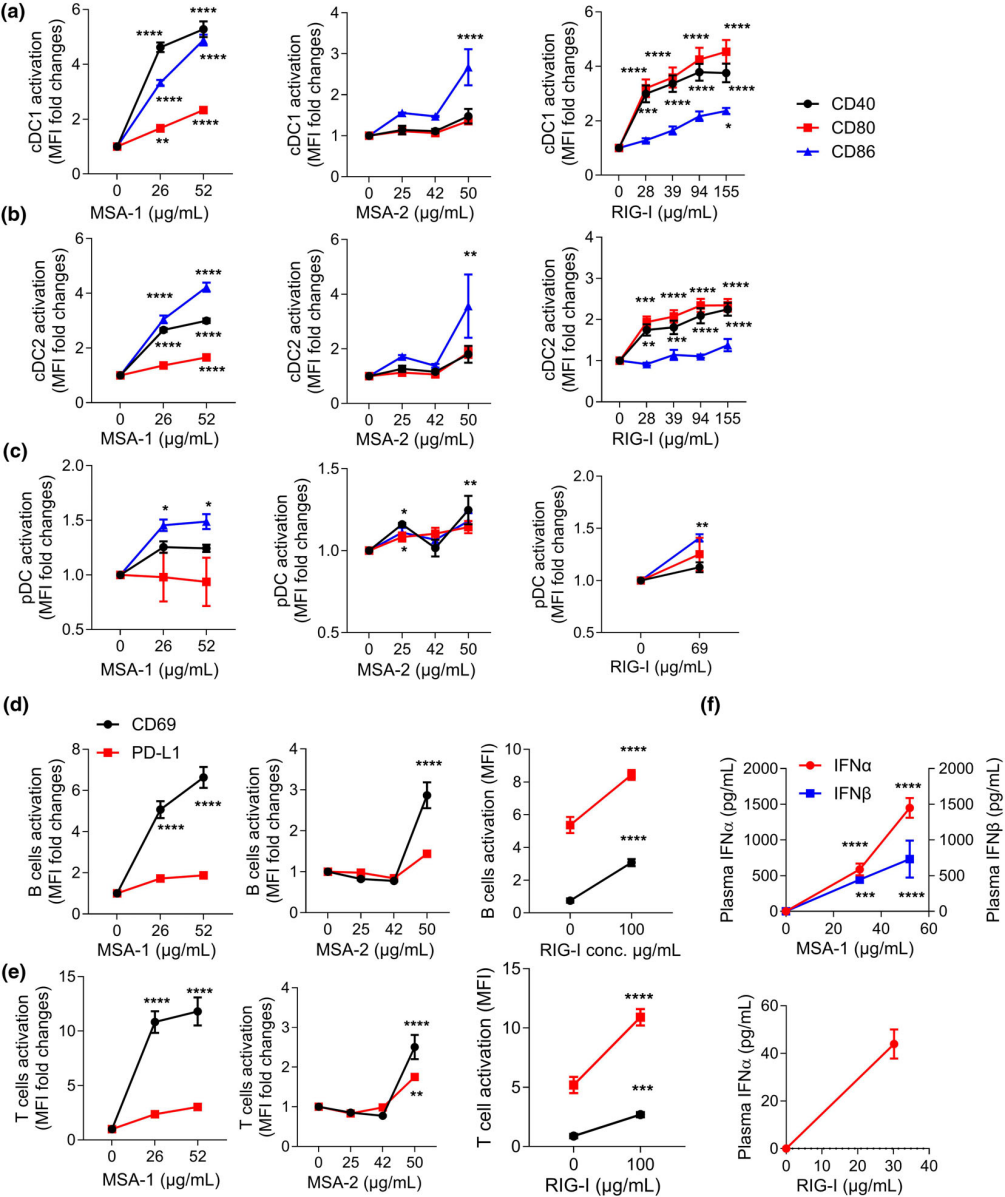
Uptake and immune stimulation by WH‐liposomes encapsulating STING or RIG‐I agonists *in vivo*. WH‐liposomes (100 μL) encapsulating concentrations of MSA‐1, MSA‐2 or RIG‐I were injected i.v. into naïve C57/BL6 mice (*n* = 2–4 per group). After 6 h, splenocytes were stained to assess levels of CD40, CD80 and CD86 expression in cDC1 **(a)**, cDC2 **(b)** and pDC **(c)**, and PD‐L1 and CD69 in B cells **(d)** and T cells **(e)** by flow cytometry. Plasma IFN‐α and IFN‐β were measured by ELISA (f). Representative of 2 replicate experiments. Statistical analysis was performed using two‐way ANOVA, with all dose groups compared to the blank liposome group (baseline = 1) using multiple comparison tests. Data points represent mean + SEM. Significance is indicated as *P* < 0.05 (*), *P* < 0.01 (**), *P* < 0.001 (***), and *P* < 0.0001 (****).

We next encapsulated varying concentrations of the STING agonists MSA‐1 and MSA‐2 into WH‐liposomes. Whereas RIG‐I was added to the lipid phase prior to running the microfluidiser, MSA‐1 and MSA‐2 were post‐loaded into WH‐conjugated liposomes (Supplementary table 3). Six hours after liposomes encapsulating increasing concentrations of agonists (Supplementary table 3) were injected i.v., splenic DCs, B cells, T cells and plasma IFN type I levels were compared. Liposomes encapsulating STING agonist MSA‐1, at concentrations of 26–52 μg/mL, or RIG‐I agonist at concentrations of 28–155 μg/mL upregulated costimulatory/activation marker expression by cDC1, cDC2, B or T cells from 2 to 15‐fold, relative to blank liposomes. This increased as liposome agonist concentration increased for cDC1 markers and CD69 expression by B and T cells. In contrast, liposomes encapsulating STING agonist MSA‐2, at similar concentrations to MSA‐1, stimulated the same cells < 2‐fold, compared to blank liposomes (Figure 2a–e). Plasmacytoid DCs were barely activated by liposomes encapsulating STING or RIG‐I agonists (Figure 2c). Whereas liposomes encapsulating MSA‐1 induced a dose‐dependent secretion of IFN‐α and IFN‐β, liposomes encapsulating RIG‐I agonist induced a lower level of IFN‐α and no IFN‐β (Figure 2f).

**Figure 3:**
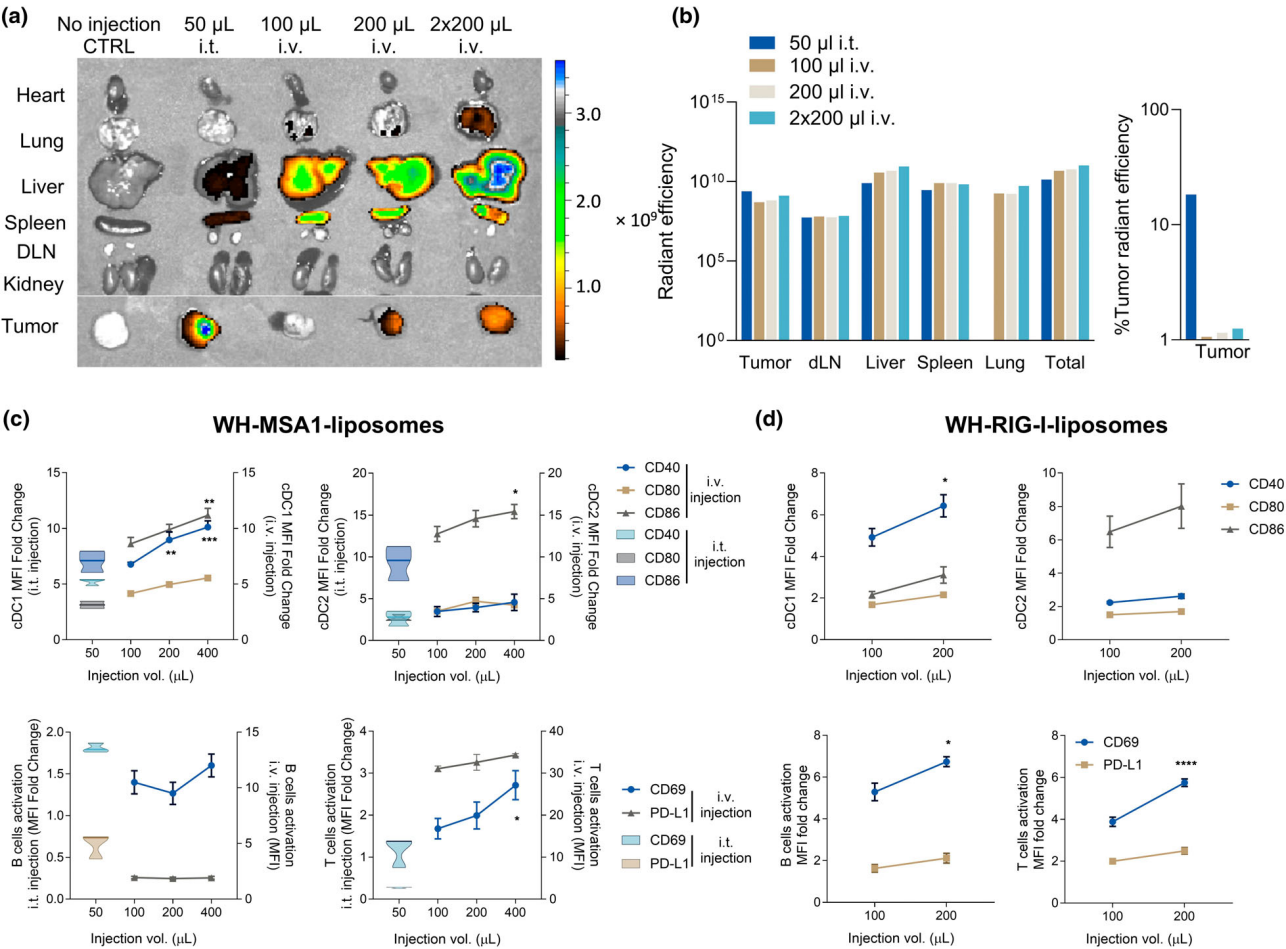
Clec9A‐STING and RIGI‐I liposomes reach tumors and activate conventional DC1. WH‐liposomes encapsulating MSA‐1 (Supplementary table 4) were administered to TC‐1 tumor bearing mice in the volumes shown. Organs were collected **(a)** and quantified **(b)** for luminescence 24 h later. Radiant efficiency is a direct measure of the total amount of liposomes accumulated and % tumor radiant efficiency indicates the proportion of the total recovered fluorescent signal located within the tumor relative to the whole body. Splenocytes were analysed by flow cytometry (*n* = 3 per group) **(c)**. WH‐liposomes encapsulating RIG‐I were administered i.v. to TC‐1 tumor‐bearing mice in the volumes shown. Splenocytes were analysed by flow cytometry (*n* = 3 per group) **(d)**. Statistical analysis used two‐way ANOVA, with all dose groups compared to the lowest dose group using multiple comparison tests. Data points represent mean + SEM. Significance is indicated as *P* < 0.05 (*), *P* < 0.01 (**), *P* < 0.001 (***), and *P* < 0.0001 (****).

To ascertain the biodistribution of liposomes after i.v. injection, we labelled WH‐liposomes encapsulating high concentrations of MSA‐1 or RIG‐I agonist, then injected volumes up to 200 μL into mice bearing subcutaneous TC‐1 tumors. We compared radiant efficiency in the tumor after i.v. injection to a 50 μL i.t. injection. To achieve higher agonist delivery to cDC1 and tumors, some liposome preparations were concentrated 2× post‐production (Supplementary table 4). The i.t. injection and 2× concentrated 200 μL liposome i.v. injection had highest radiant efficiencies in tumor (Figure 3a). Radiant efficiency also increased in lung, liver and spleen in a dose‐dependent manner (Figure 3b). After i.v. liposome administration, WH‐liposome‐encapsulated MSA‐1 and RIG‐I agonist activated splenic cDC1, cDC2, B and T cells dose dependently. Both agonists activated cDC1 more strongly than cDC2, and MSA‐1 activated T‐cell CD69 more effectively than RIG‐I (Figure 3c and d).

**Figure 4:**
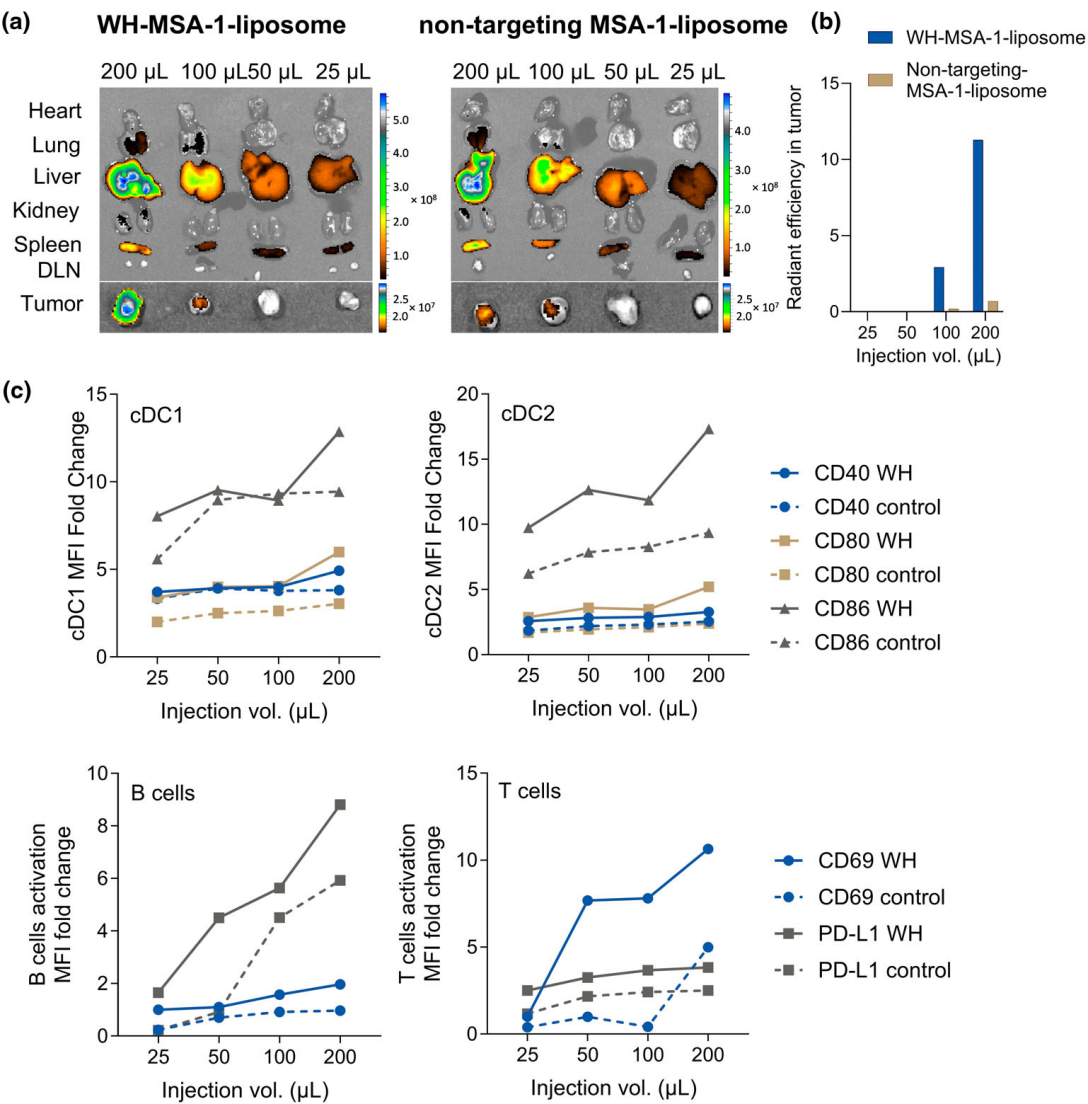
Functionalising STING liposomes with Clec9A‐targeting WH peptide to improve tumor uptake and activation of conventional DCs. WH‐liposomes or non‐targeting control liposomes encapsulating MSA‐1 were administered i.v. to TC1 tumor‐bearing mice in the volumes shown. Organs were collected **(a)** and quantified **(b)** for luminescence 24 h later. Splenocytes were analysed by flow cytometry for activation and exhaustion markers **(c)**. Data are from a pilot experiment to identify the volume of WH‐MSA‐1 liposome able to activate immune cells *in vivo*. The data show fold changes in activation markers for liposome‐treated relative to untreated mice.

In an exploratory pilot assessment, we observed that functionalising MSA‐1 liposomes with Clec9A‐targeting WH peptide increased liposome uptake in TC‐1 tumor after i.v. administration (Figure 4a and b) and increased the activation of splenic immune cells, compared to control MSA‐1 liposomes functionalised with polylysine (Figure 4c). As little as 25 μL WH‐MSA‐1 liposomes appeared to be sufficient to stimulate splenic cDC1 and cDC2, and 50 μL for B and T cell activation above untargeted liposomes, with a dose‐dependent increase in activation. Thus after i.v. administration, intra‐tumoral distribution and cDC1 activation were most prominent with 2× concentrated 200 μL WH‐liposomes, encapsulating ~100 μg/mL MSA‐1.

**Figure 5:**
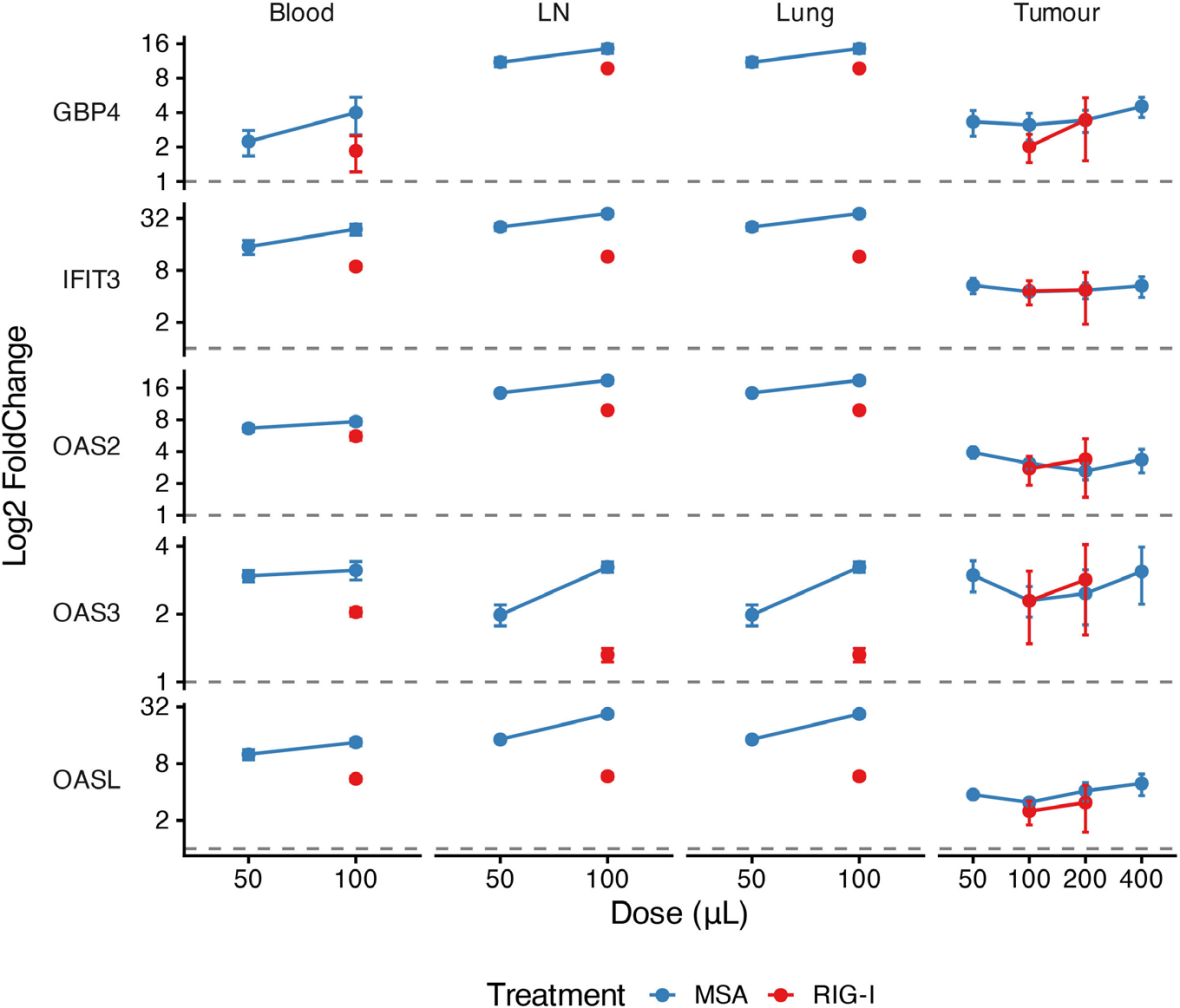
Intravenous administration of Clec9A‐functionalised STING or RIG‐I liposomes activates IFN‐dependent pathways in dLN, lung, blood and tumor. WH‐liposomes encapsulating MSA‐1 or RIG‐I or empty liposomes were administered to B16F10 tumor‐bearing mice in the volumes shown. RNA was prepared from organs as shown then analysed using a custom Nanostring nCounter IFNα/β Codeset per manufacturer's instructions. Individual expression profiles of the five most highly induced interferon‐stimulated genes (ISGs: GBP4, IFIT3, OAS2, OAS3, OASL) are shown across blood, tumor draining lymph node (LN), lung and tumor. Genes were selected based on fold‐change over empty liposome control in tumor tissue at the highest MSA dose. Each panel shows dose–response curves for MSA (blue) and RIG‐I agonist (red). Data are presented as mean ± SEM (*n* = 3 or 4 biological replicates per group). Normalised expression of the top five tumor‐responsive ISGs is presented as fold‐change relative to empty liposome control on a log2 scale.

To determine whether and where a type 1 IFN transcriptional signature was expressed after i.v. liposomes, we analysed tissue RNA for IFN‐induced genes by Nanostring 24 h after injection of 100 μL blank WH‐liposomes or 100 or 50 μL of MSA‐1 or RIG‐I WH‐liposomes into TC‐1 tumor‐bearing mice, compared to a positive control of DC stimulated with IFNα *ex vivo*. Normalised expression of the top 5 IFN‐induced genes, OAS2, OAS3, OASL, GBP4 and IFIT3 are represented as fold‐change relative to empty liposome vehicle control (Figure 5, full heatmap in Supplementary figure 2). Genes were selected based on fold‐change over empty liposome vehicle control in tumor tissue at the highest MSA dose. The individual gene plots demonstrate that the ISG response is driven by coordinated upregulation of multiple independent genes and that there is consistent interferon pathway activation in blood, spleen, lung, tumor draining LN and tumor microenvironment, with each gene displaying consistent organ‐specific expression but distinct dose‐relationship. The dose response in the tumor suggests efficient gene induction already at a 50 μL i.v. dose of MSA liposomes, consistent with the targeted mode of action of the liposomes while extra‐tumoral ISG expression increased dose dependently, consistent with greater systemic effects as the dose increased. Changes after i.t. RIG‐I liposomes were more variable, consistent with targeting variability with this mode of administration. While the systemic induction of ISGs was greater after i.v. MSA1 than i.t. RIG‐I WH liposomes, there was better or equivalent, albeit low level, ISG induction by i.v. MSA‐1 than i.t. RIG‐I liposomes in the tumor, consistent with effective i.v. targeting of a solid tumor with an immunosuppressive micro‐environment (Figure 5). These data confirm that the tumor ISG response is biologically significant.

**Figure 6:**
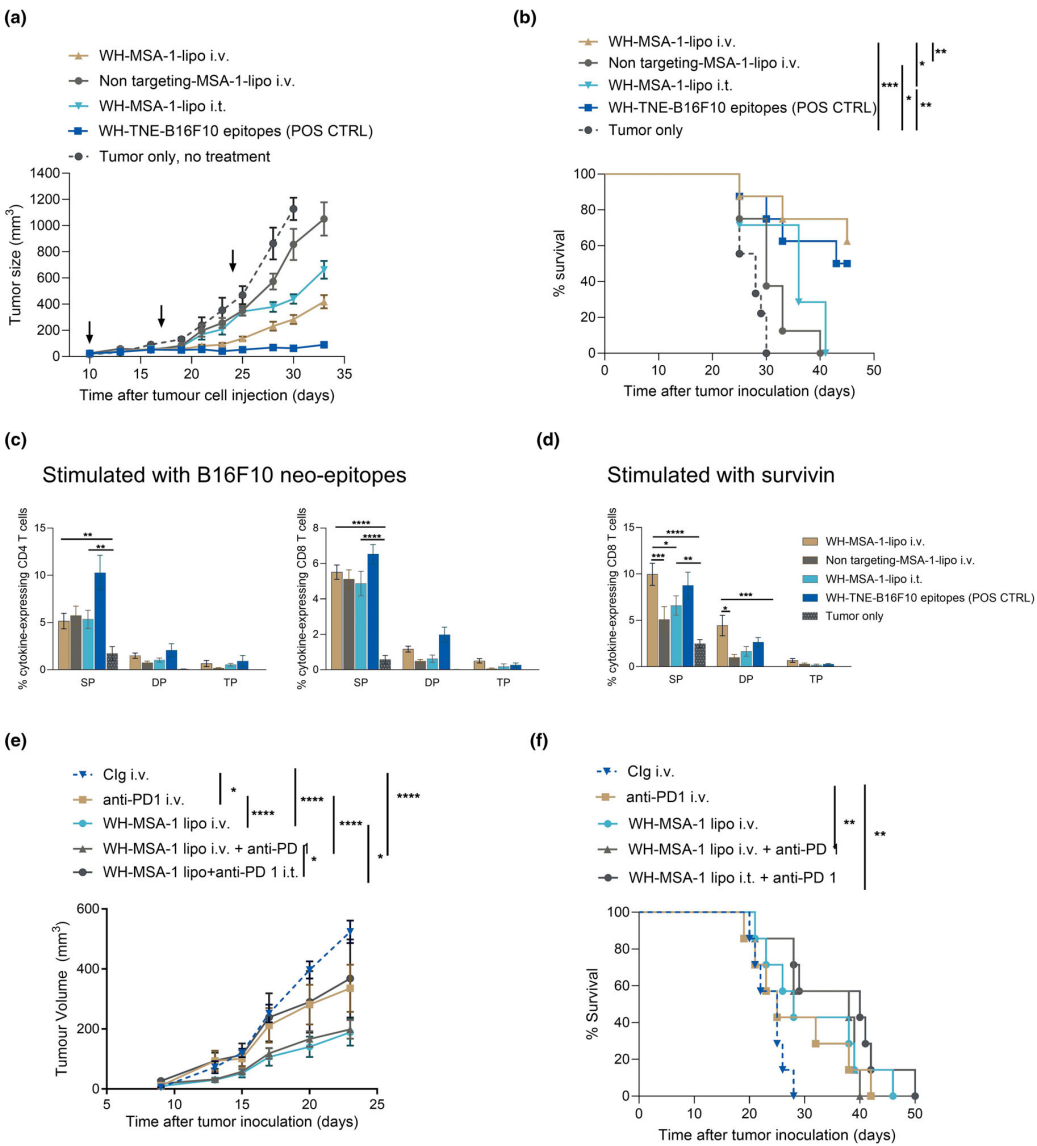
Intravenous Clec9A‐STING liposomes suppress B16 melanoma growth and prolong survival. WH‐liposomes encapsulating 5 μg MSA‐1 i.v. (2 × 200 μL) or 2.5 μg i.t. (50 μL), nontargeted 5 μg MSA‐1 liposomes i.v., WH‐TNE encapsulating B16F10 CD4^+^ and CD8^+^ epitopes i.v. or no treatment were administered weekly to B16 tumor‐bearing mice, beginning at day 10 after tumor inoculation. Tumor volumes **(a)** and survival **(b)** were measured twice weekly for 5–6 weeks. *P < 0.05, **P < 0.01, ***P < 0.001 (survival analysis). Whole blood was stimulated with pooled B16F10 CD4^+^ and CD8^+^ epitopes **(c)** or survivin peptide **(d)** *ex vivo* and CD4^+^ and CD8^+^ T cell cytokines were analysed by flow cytometry 48 h later. The frequencies of IFN‐γ, IL‐2, and TNF‐α single (SP), double (DP), or triple positive (TP) CD4+ and CD8+ T cells are shown. Data represent means from 5 mice over 2 experiments. Data points represent mean + SEM. *P < 0.05, **P < 0.01, ***P < 0.001, ****P < 0.0001. (mixed effect model). **(e)** WH‐liposomes encapsulating 5 μg MSA‐1 i.v. (2 × 200 μL), anti‐PD1 (muDX400) or isotype control antibody (CIg) were administered i.v. or i.t. weekly as shown to B16 tumor‐bearing mice (*n* = 8 over 2 experiments), beginning at day 10 after tumor inoculation. Tumor volumes and survival **(f)** were measured twice weekly for 3–7 weeks. *P < 0.05, **P < 0.01, ***P < 0.001, ****P < 0.0001 (mixed effect model tumor volume, log‐rank test survival). Data points represent mean + SEM.

To determine their potential for tumor immunotherapy, we inoculated mice s.c. with B16F10 melanoma cells and 10 days later administered WH‐MSA‐1 liposomes i.v. (2× 200 μL) or i.t. (50 μL) weekly. B16F10 melanoma has low immunogenicity in mice and is poorly responsive to anti‐PD1, while Clec9A‐targeted TNE encapsulating B16F10 neoepitopes strongly enhance immunogenicity and tumor control.^24^ We compared the tumor growth and survival of mice treated with WH‐TNE, or i.v. WH‐ or untargeted MSA‐1 liposomes. Similar to WH‐TNE‐neoepitopes, WH‐MSA‐1 liposomes i.v. significantly suppressed tumor growth and improved survival relative to no treatment, i.v. untargeted MSA‐1 liposomes or i.t. WH‐MSA‐1 liposomes (Figure 6a and b). Significantly more polyfunctional CD8^+^ T cells were expanded in blood in response to B16F10 neo‐epitopes and the tumor‐associated antigen, survivin, after i.v. WH‐MSA‐1 liposomes relative to untreated mice. WH‐MSA‐1 liposomes also expanded more cytokine‐producing CD4^+^ and CD8^+^ T cells in response to neo‐epitopes than untreated mice (Figure 6c and d). No sign of systemic toxicity was recorded in mice injected with WH‐ or untargeted MSA‐1 liposomes. These data demonstrate that targeting cDC1 with the STING agonist MSA1 i.v. expands tumor‐specific CD4^+^ and CD8^+^ T cells and enhances survival from B16F10 melanoma. Consistent with the poor response of B16‐F10 melanoma to anti‐PD1, anti‐PD1 mAb DX‐40 alone had minimal impact on tumor growth relative to isotype control mAb and it did not suppress growth further when added to i.v. WH‐MSA‐1 liposomes (Figure 6e and f).

**Figure 7:**
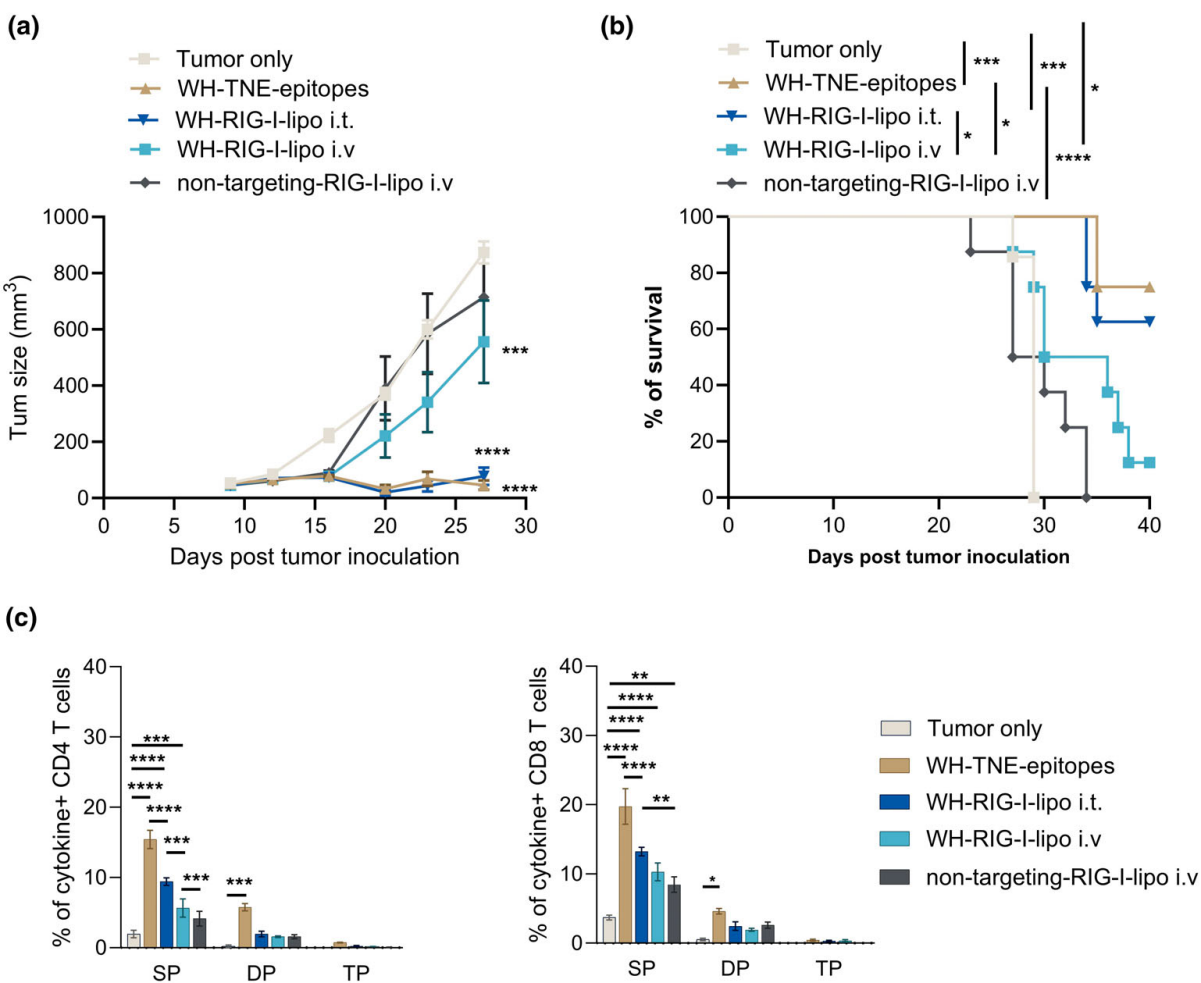
Intratumoral Clec9A‐RIG‐I liposomes suppress B16 melanoma growth and prolong survival. WH‐liposomes encapsulating RIGI‐I i.v. or i.t., non‐targeted RIG‐I liposomes i.v., WH‐TNE encapsulating B16F10 CD4^+^ and CD8^+^ epitopes i.v. or no treatment were administered weekly to B16 tumor‐bearing mice (*n* = 8 over 2 experiments), beginning at day 10 after tumor inoculation. Tumor volumes **(a)** and survival **(b)** were measured twice weekly for 5–6 weeks. Data points represent mean + SEM. *P < 0.05, ***P < 0.001, ****P < 0.0001 (mixed effect model tumor volume, log‐rank test survival). Whole blood were stimulated with pooled B16F10 CD4^+^ and CD8^+^ epitopes **(c)** *ex vivo* and CD4^+^ and CD8^+^ T cell cytokines were analysed by flow cytometry 48 h later as for Figure 5e. Data represent means from 5 mice over 2 experiments. Data points represent mean + SEM. **P < 0.01, ***P < 0.001, ****P < 0.0001 (mixed effect model).

In contrast to WH‐MSA‐1 liposomes, WH‐RIG‐I liposomes administered s.c. induced low levels of cDC1 activation and type 1 IFN production *in vivo*. To determine the potential of WH‐RIG‐I liposomes for tumor immunotherapy, we inoculated mice s.c. with B16F10 melanoma cells then 10 days later, administered WH‐RIG‐I liposomes i.v. or i.t. weekly. Whereas WH‐RIG‐I liposomes i.v. did not reduce tumor growth or improve survival relative to untreated mice, i.t. WH‐RIG‐I liposomes suppressed tumor growth and improved survival to a similar extent as WH‐TNE with no evidence of local or systemic toxicity (Figure 7a and b). Significantly more cytokine‐producing CD4^+^ and CD8^+^ T cells in response to neo‐epitopes and survivin were expanded by all treatments relative to untreated mice (Figure 7c). These data demonstrate that i.t. targeting of cDC1 with RIG‐I is safe and successfully expands tumor‐specific CD4^+^ and CD8^+^ T cells, leading to enhanced survival from the poorly immunogenic B16F10 melanoma.

## Discussion

Targeting approaches, including conjugation of antigen to Clec9A‐specific monoclonal antibodies and functionalising nanoparticles with the Clec9A‐specific WH peptide, have been shown to be safe and efficient Clec9A‐targeting tools that improve anti‐viral vaccine responses and anti‐tumor immunity.^26^, ^27^, ^28^, ^29^ In the current studies, we designed liposomes to deliver STING or RIG‐I agonists to cDC1 via Clec9A targeting with WH. Liposomes have the advantage of protecting the agonist cargo from enzymatic degradation, and Clec9A targeting also facilitates intracellular agonist delivery to the cytosol, where STING is located. cDC1s are highly conserved across species and represent a critical bridge for cross‐presenting tumor antigens to T cells in both mice and human. We show that i.v. Clec9A‐STING liposomes are preferentially taken up by cDC1 and pDCs that both express Clec9A in mice. By incorporating the potent STING agonist, MSA‐1, Clec9A‐liposomes delivered type 1‐IFN‐mediated activation signalling to splenic DCs after i.v. administration and at a lower level after i.t. administration. Widespread expression of a type 1 IFN transcriptomic signature was observed in blood, tumor draining lymph node, lung and tumor, associated with upregulation of interferon‐stimulated genes expected to activate antigen presenting cells and chemo‐attract activated T cells, NK cells and monocytes to the tumor.^30^ Interestingly, the ISG dose response suggested efficient gene induction with a low i.v. dose of MSA liposomes, consistent with the targeted mode of action of the liposomes, while there was a more systemic dose‐dependent increase in ISG. Both i.v. Clec9A‐STING liposomes and i.t. Clec9A‐RIG‐I liposomes expanded tumor antigen‐specific polyfunctional CD4 and CD8 T cells and controlled tumor growth with enhanced survival. In contrast, these outcomes were less effective with i.t. Clec9A‐STING liposomes, i.v. Clec9A‐RIG‐I liposomes, or antiPD‐1 mAb alone.

In the current studies, the type 1 IFN signature in blood, lung, tumor and tumor draining lymph node corresponded to the induction of IFN‐α in plasma after i.v. administration of WH‐MSA1 or WH‐RIG‐I liposomes. pDCs, which took up WH‐liposomes, produce large amounts of IFN‐α, when signalled through the c‐GAS‐STING pathway.^3^ Tumor‐derived exosomes were shown to transport tumor dsDNA into the cytosol of DCs to activate this pathway.^31^ Clec9A‐MSA‐1 or RIG‐I liposomes, which have similar properties to exosomes, also transported agonists intracellularly to activate c‐GAS‐STING or RIG‐I, resulting in a systemic IFN‐mediated cellular activation *in vivo*. Not only were cDC1 activated, but in the tumor immunotherapy setting, survivin and neo‐epitope specific CD4^+^ and CD8^+^ T cells were expanded by WH‐MSA‐1 and RIG‐I liposomes, indicating that multiple bystander tumor epitopes were presented for adaptive immunity engagement. In the B16F10 melanoma model, these liposomes administered from Day 10 were more effective than anti‐PD‐1. Furthermore, there was no enhancement of liposome efficacy with anti‐PD‐1, likely because of induction of similar mechanistic pathways, which was more rapid with MSA‐1 liposomes targeting tumor Clec9A than with anti‐PD‐1.^14^ It is possible that immune checkpoint blockade could be more effective if administered at later time points, to counteract the tumor‐induced exhaustion of tumor‐specific T cells previously induced by STING or RIG‐I agonists.

Although neo‐antigen identification has become feasible and affordable by advances in massively parallel sequencing, the formulation and immunogenic delivery of neo‐epitopes in a timely personalised manner for effective cancer vaccination remains a major challenge. By exploiting a nanoparticulate strategy to target Clec9A^+^ DC1 and pDC to activate their capacity for presentation of tumor antigens processed *in situ*, and for type 1 IFN production respectively, we show that the bed of a cold tumor can be rapidly activated to a ‘hot’ environment with initial expansion of anti‐tumor immune response and suppression of tumor growth, prior to tumor‐specific immunotherapy, for example with tailored nanoemulsions.^24^ We created a translatable, scalable Clec9A‐liposome formulation using semi‐synthetic lipids, PEG and WH targeting peptide in view of the established clinical track record of liposomes. We used microfluidic technology as it has the advantage of rapid laboratory optimisation, as we show here, with a high level of process control and reproducibility, and relative ease of scale up for product manufacture.^32^

Versatile cancer vaccines are a much‐needed therapeutic strategy in cancer immunotherapy. Clec9A‐STING liposomes boost tumor antigen immunogenicity via type 1 IFN induction, DC antigen cross‐presentation and polyfunctional tumor‐specific CD4^+^ and CD8^+^ T‐cell expansion. They can be produced at scale and administered i.v., avoiding i.t. administration, and have potent efficacy in the poorly immunogenic B16F10 cutaneous melanoma preclinical model. Clec9A‐STING liposomes represent a versatile, translatable immunotherapy strategy for poorly immunogenic cancers.

## Methods

### Materials, mice

WH peptide (WPRFHSSVFHTHGGGK),^33^ CT26 and B16F10 neoantigen epitopes^24^ (> 95% purity) were synthesised by GL Biochem (Shanghai, China). 1,2‐dipalmitoyl‐sn‐glycero‐3‐phosphocholine (DPPC) was purchased from Avanti Polar Lipids, Inc. (Alabaster, AL) (purity > 99%), cholesterol from Sigma‐Aldrich, and DSPE‐PEG‐NHS (MW 5000, PDI < 1.08, purity > 95%) from Nanocs. MSA‐1 and MSA‐2 STING agonists and RIG‐I agonist were synthesised at MSD, according to a published method.^34^ Anti‐PD1 (muDX400) was generated by MSD and is a murinised version of a rat anti–mouse PD1 Ab with a mutated D265A mouse IgG1 Fc. It was dosed at 5 mg/kg. The isotype control Ab mouse anti‐hexon IgG1 27F11 was generated by MSD. AF488‐anti‐CD3 (17A2), PE/Cy7‐anti‐CD4 (RM4‐4), APC/Cy7‐anti‐CD8 (53.67), PE/Cy7‐anti‐CD11c (N418), APC‐anti‐CD317 (927), PE‐anti‐CD45.2 (104), PerCP/Cy5.5‐anti‐I‐A/I‐E (M5/114.15.2), PerCP/Cy5.5‐anti‐CD40 (3/23), PE‐anti‐CD80 (16‐10A1), FITC‐anti‐CD86 (GL‐1), APC‐anti‐CD4 (RM4‐5), PerCP/Cy5.5‐anti‐CD45 (I3/2.3), PE/Cy7‐anti‐CD44 (IM7), PE‐anti‐PD1 (29F.1A12), APC/Cy7‐anti‐CD4 (RM4‐5), APC/Cy7‐anti‐CD11b (M1/70), FITC‐anti‐F4/80 (BM8), APC‐anti‐CD206 (C068C2), AF700‐anti‐IFNγ (XMB1.2), PE‐anti‐IL2 (JES6‐5H4) and AF647‐anti‐TNF (MP6‐XT22) were from Biolegend. Anti‐mouse LAMP‐1 (1D4B) and anti‐mouse EEA1 (14/EEA1) were from BD Biosciences. Tetramethylindocarbocyanine Perchlorate (DiI) and LIVE/DEAD® Fixable Aqua Dead cell stain were from Molecular Probes. C57BL/6 mice were purchased from the Animal Resources Centre (Perth, WA, Australia). Batf3^−/−^ mice were purchased from Jackson Laboratories (Bar Harbor, ME, USA) and bred at The University of Queensland under specific pathogen‐free conditions.

### Liposome preparation

Liposome formulations were prepared using the benchtop NanoAssemblr™ instrument (NanoAssemblrTM, Precision Nano‐Systems Inc.). The lipid phase (DPPC, cholesterol, DSPE‐PEG_5000_‐NHS) (molar ratio—34:3.5:25:0.6) and aqueous buffer (10 mM Hepes, pH 7.4) were used to prepare the liposomes. Non‐entrapped material was separated by overnight dialysis using a 10 kDa dialysis membrane (Thermo Fisher Scientific, USA). For the preparation of targeted liposomes, either pre‐conjugated DSPE‐PEG_5000_‐WH peptide was added to the lipid phase during liposome preparation, or the peptide was post‐conjugated to DSPE‐PEG_5000_NHS‐liposomes. For post‐conjugated liposomes, unconjugated peptide was separated from WH‐conjugated liposomes by overnight dialysis. Non‐targeting liposomes were prepared following the same procedure, but WH‐peptide was substituted with poly‐lysine. For RIG‐I‐loaded liposomes, 100 μg/mL RIG‐I was added to the aqueous phase prior to running them on the microfluidiser. For STING‐loaded liposomes, 75 μg/mL MSA‐1 was post‐loaded to WH‐conjugated liposomes. Unencapsulated MSA‐1 was separated by overnight dialysis. For the preparation of 2× concentrated formulation, centrifugal filter tubes (Merck Millipore, USA) were used. The liposomes were stored at 2–8°C until further analysis.

### Liposome characterisation

Dynamic light scattering (DLS) was used to report the intensity mean diameter (z‐average) and the polydispersity of all liposome formulations (Malvern Zetasizer Nano‐ZS (Malvern Instruments, Worcestershire., UK)). The measurements of vesicle size and polydispersity were carried out at 25°C in Hepes buffer ([1:10], 1 mM, pH 7.4). Zeta potential was measured in MilliQ water pH 7.4 using the Malvern Zetasizer Nano‐ZS (Malvern Instruments, Worcestershire., UK). WH peptide concentration and encapsulated agonist concentrations were measured using HPLC. All measurements were undertaken in triplicate.

### Preparation of single‐cell suspensions

Spleens and lymph nodes were harvested and placed in ice‐cold PBS. Tissues were mechanically minced and then enzymatically digested with 1 mg/mL Collagenase Type IV and 0.1 mg/mL DNase I for 30 min at 37°C. The resulting cell suspensions were passed through a 70 μm cell strainer, followed by ACK lysis buffer (0.15 M NH_4_Cl, 1 mM KHCO_3_, 0.1 mM EDTA). Cells were washed and resuspended in FACS buffer (PBS containing 2% FBS and 2 mM EDTA) at 4°C for antibody staining.

### Flow cytometry and confocal microscopy

For liposome *in vivo* distribution experiments, single‐cell suspensions were prepared from spleens and stained for I‐A/I‐E, CD11c, CD317, CD4, and CD8 surface markers. Dead cells were excluded using Live/Dead Aqua stain. pDCs were gated as IA/IE^+^CD11c^int^CD317^+^. CD8+ cDCs were gated as IA/IE^+^CD11c^hi^CD8^+^ and CD8‐ cDCs as I‐A/I‐E^+^CD11c^hi^CD8^−^. All cell suspensions were first incubated on ice with a 1:1000 dilution of Live/Dead Aqua for 15 min and then with rat anti‐mouse FcγIII/II receptor (CD16/CD32) blocking antibodies (4 μg mL^−1^, BD Inc.) for 15 min on ice. Samples were acquired on a Beckman Coulter CytoFLEX and analysed with BD KALUZA (Figures 1, 2, 3, 4) or FlowJo (Figures 6 and 7) software.

### *In vivo* biodistribution and immune activation with liposomes

For biodistribution studies, 100 μL DiI‐labelled liposomes was injected i.v. to TC‐1 tumor bearing C57BL/6 mice. Mice were imaged using IVIS 6 h later, and organs removed to compare fluorescence intensity. Splenic DCs were stained to identify uptake of liposomes by DC subsets by flow cytometry. For immune activation studies, 100 μL liposomes was injected i.v. to C57BL/6 mice. Splenic DCs, B cells and T cells were stained for cell surface activation markers by flow cytometry. Plasma was analysed for IFN‐α and IFN‐β by ELISA using the VeriKine Mouse IFN Alpha ELISA Kit (Pbl, NJ, USA). Liver, spleen, tumor, blood, kidney, heart and tumor draining lymph node were snap frozen for transcriptomic analysis by Nanostring.

### Nanostring

WH‐liposomes encapsulating MSA‐1 or RIG‐I were administered to B16 tumor‐bearing mice in the volumes shown. RNA was prepared from organs as shown and from DCs transfected with a negative control RNA (negative control) or with a RIG‐I agonist (positive control) then analysed using a custom Nanostring nCounter IFNα/β Codeset per manufacturer's instructions.

### Mouse models

For therapeutic experiments in the melanoma model, C57BL/6 mice were inoculated s.c. with 2.5 × 10^4^ B16F10 melanoma cells into the flank and randomly distributed into treatment groups. Tumor volumes were measured with callipers and calculated using the formula (A × B^2^)/2 (A as the largest and B as the smallest diameter of the tumor). Mice received 4 weekly i.v. or i.t. doses of WH‐ or non‐targeting‐liposomes encapsulating MSA‐1 or RIG‐I, or i.v. WH‐TNE encapsulating 6 neo‐epitopes (10 μg of mutated epitopes per mouse),^12^ commencing 10 days after tumor inoculation.

### Splenocyte stimulation for T‐cell polyfunctionality

Blood and spleens were collected at different time points. Spleens were finely chopped, and a single‐cell suspension was obtained by passing the sample through a 70 μm nylon strainer. Erythrocytes were eliminated with ACK lysis buffer. Samples were then washed and resuspended in RPMI‐10. Cytokine production in T cells was measured by flow cytometry and intracellular cytokine staining after *in vitro* stimulation of whole blood samples with tumor‐associated antigen survivin_20‐28_ peptide or a pool of B16F10 neo‐epitopes for 5 h to determine the frequency of IFN‐γ, IL‐2 and TNF‐α single (SP), double (DP), or triple positive (TP) CD4^+^ and CD8^+^ T cells.^35^

### Statistical analyses

Results are presented as mean with SEM of each group. Data were statistically analysed by ordinary one‐way or, where appropriate, two‐way ANOVA and Tukey's multiple comparisons test, using the Prism GraphPad software. *P < 0.05; **P < 0.01; ***P < 0.001; ****P < 0.0001. Survival analyses used the Mantel–Cox log‐rank test. A heatmap was generated using the pheatmap package in R. *Z*‐scores were calculated based on normalised counts obtained from NanoString gene expression data to facilitate relative comparison across genes.

### Study approval

The animal experiments were approved by Animal Ethics Committees (AEC) of the University of Queensland, Brisbane, Australia.

## Author contributions

**Gretchen A Baltus:** Conceptualization; writing – review and editing; project administration; supervision; resources. **Karyn van de Mark:** Investigation; writing – review and editing. **Yulia Zybina:** Investigation; writing – review and editing. **Thais Aragao‐Horoiwa:** Investigation; methodology; writing – review and editing. **Wendy Blumenschein:** Investigation; writing – review and editing. **Meghna Talekar:** Conceptualization; methodology; investigation; writing – review and editing; supervision. **Guangzu Zhao:** Investigation; writing – review and editing. **Amy Cameron:** Investigation; writing – review and editing. **Wittaya Suwakulsiri:** Investigation; writing – review and editing; visualization. **Bijun Zeng:** Methodology; formal analysis; investigation; writing – original draft. **Riccardo Dolcetti:** Conceptualization; funding acquisition; writing – review and editing; supervision; resources. **Ranjeny Thomas:** Supervision; resources; project administration; funding acquisition; writing – original draft.

## Conflict of interest

YZ, WB, KVDM, HF, AD, and GAB were all employees of Merck Sharp & Dohme LLC, a subsidiary of Merck & Co., Inc., Rahway, NJ, USA (MSD), during the conduct of this work. The work was funded by a research contract with MSD.

## Supporting information


Supplementary table 1
Supplementary table 2
Supplementary table 3
Supplementary table 4
Supplementary figure 1
Supplementary figure 2

## Data Availability

The data that support the findings of this study are available from the corresponding author upon reasonable request.
