# Supplementary material for: Control of poorly immunogenic tumors with systemic STING agonist‐loaded liposomes targeting cross‐presenting dendritic cells
Source: Clin Transl Immunology. 2026 Jun 1;15(6):e70110. doi: 10.1002/cti2.70110 (PMC13238515; doi:10.1002/cti2.70110)

**Supplementary data**

**Supplementary Table 1. Liposome characteristics according to flow rate and WH/PEG%**

FPA liposomes were prepared with four different flow rates and NHS-PEG5000-DSPE percentages. The liposome characteristics are indicated.

| **Sample** | **Z average size (nm)** | **PDI** | **Zeta potential (mV)** |
| --- | --- | --- | --- |
| DPPC/Chol 6 mL/min | 178.9 | 0.194 | -21.1 |
| DPPC/Chol 12 mL/min | 169.6 | 0.187 | -21.7 |
| DPPC/Chol 24 mL/min | 151.3 | 0.183 | -20.9 |
| DPPC/Chol 36 mL/min | 161.8 | 0.199 | -20.0 |

| **Sample** | **Z average size (nm)** | **PDI** | **Zeta potential (mV)** |
| --- | --- | --- | --- |
| DPPC/Chol | 168.4 | 0.224 | -22.8 |
| DPPC/Chol/PEG-WH 0.15% | 158.6 | 0.183 | -23.9 |
| DPPC/Chol/PEG-WH 0.25% | 192.0 | 0.266 | -22.7 |
| DPPC/Chol/PEG-WH 0.50% | 168.9 | 0.197 | -21.2 |

**Supplementary Table 2. Liposome binding by splenic DC subsets according to WH/PEG%**

DiI-labelled WH-liposomes prepared with varying concentrations of PEG were incubated with splenocytes for 1h on ice, then DiI+ MHC II+CD11c+CD8+ cDC1, MHC II+CD11c+CD8- cDC2 and B220+CD137+ pDC were quantified by flow cytometry.

| **Sample** | **DiI+ cDC1** | **DiI+ cDC2** | **DiI+ pDC** |
| --- | --- | --- | --- |
| DPPC/Chol | 2.42 | 7.58 | 1.29 |
| DPPC/Chol/PEG-WH 0.15% | 6.26 | 8.21 | 3.79 |
| DPPC/Chol/PEG-WH 0.25% | 9.47 | 6.57 | 2.52 |
| DPPC/Chol/PEG-WH 0.50% | 9.05 | 14.94 | 4.23 |

**Supplementary Table 3. Characteristics of WH liposomes encapsulating STING and RIG-I agonists**

| **Liposome, loading (μg/mL)** | **Size (nm)** | **Charge (mV)** | **PDI** | **Drug concentration (μg/mL)** |
| --- | --- | --- | --- | --- |
| WH empty | 126.7 | -18.5 | 0.077 |  |
| WH MSA-1 50 | 129.0 | -20.4 | 0.124 | 31 |
| WH MSA-1 100 | 132.4 | -19.2 | 0.130 | 52 |
| WH MSA-2 200 | 101.1 | -43 | 0.272 | 41 |
| WH RIG-I | 119.9 | -27 | 0.093 | 67 |
| WH RIG-I 28 | 116 | 0.132 | -20.3 | 28 |
| WH RIG-I 39 | 132 | 0.132 | -14.7 | 39 |
| WH RIG-I 94 | 173 | 0.139 | -20.4 | 94 |
| WH RIG-I 155 | 110 | 0.188 | -20.2 | 155 |

**Supplementary Table 4. Characteristics of WH-liposomes injected into mice bearing TC-1 or B16F10 tumors**

| **Liposome** | **Size (nm)** | **Charge (mV)** | **PDI** | **Drug concentration (μg/mL)** | **Phospholipid concentration (mg/mL)** |
| --- | --- | --- | --- | --- | --- |
| WH MSA-1 1x | 127.6 | -20.8 | 0.107 | 48 | 1.2 |
| WH MSA-1 2x | 136.4 | -26.8 | 0.105 | 48 | 3.4 |
| WH RIG-I | 119.9 | -27 | 0.093 | 67 | 0.81 |

Supplementary Figure 1. Gating strategies

A. Dendritic cell gating for liposome uptake experiments in vitro (Supplementary Table 1).


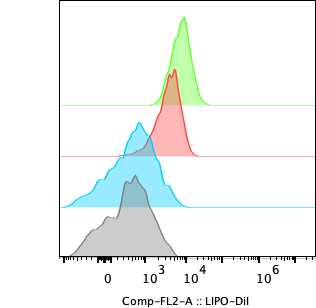

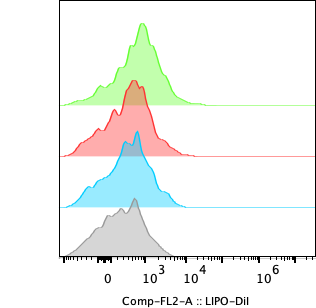

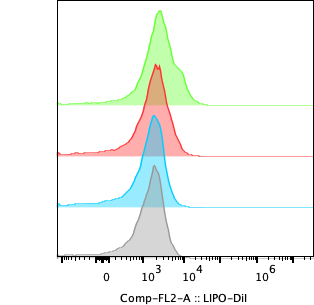


No liposome CTRL

WH-liposome 0.15%

WH-liposome 0.25%

WH-liposome 0. 5%

cDC1

cDC2

pDC

DiI-liposome intensity


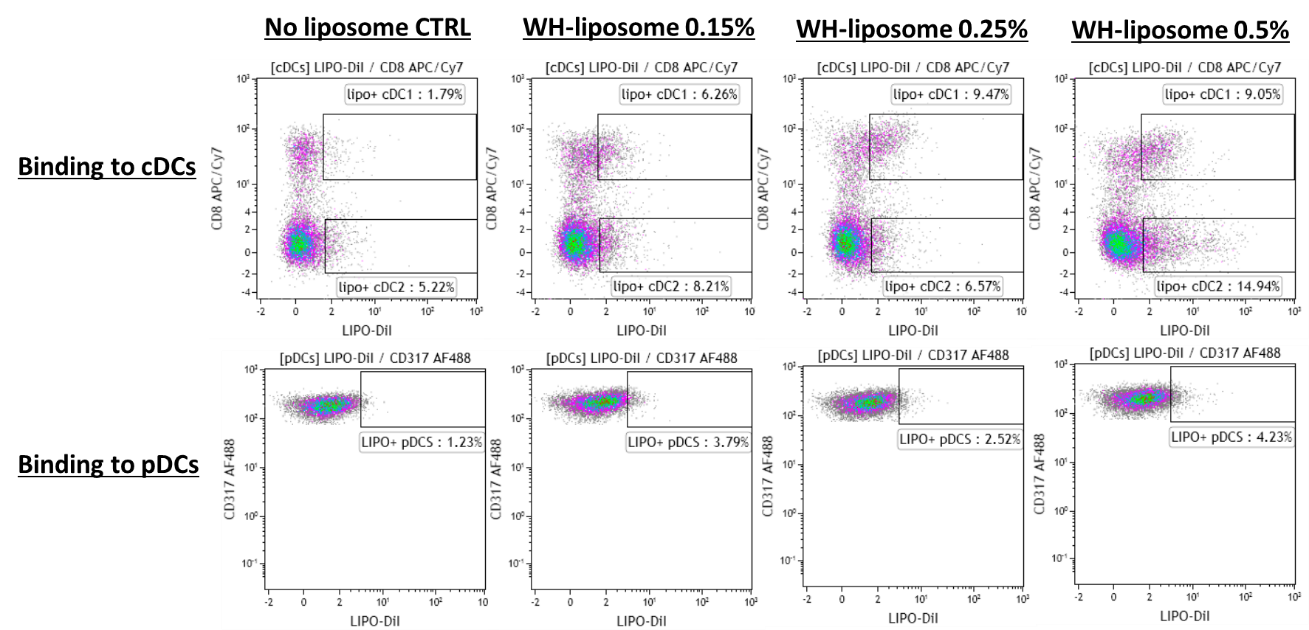


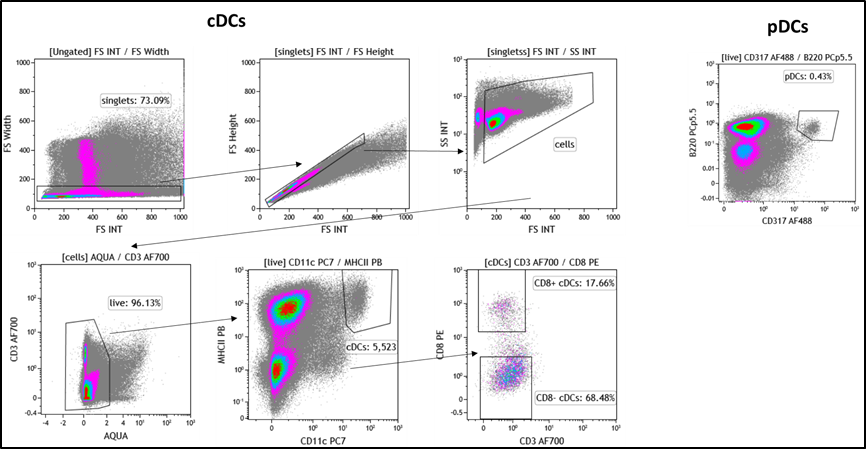
B. Dendritic cell gating for liposome uptake experiments *in vivo* (Figure 1).


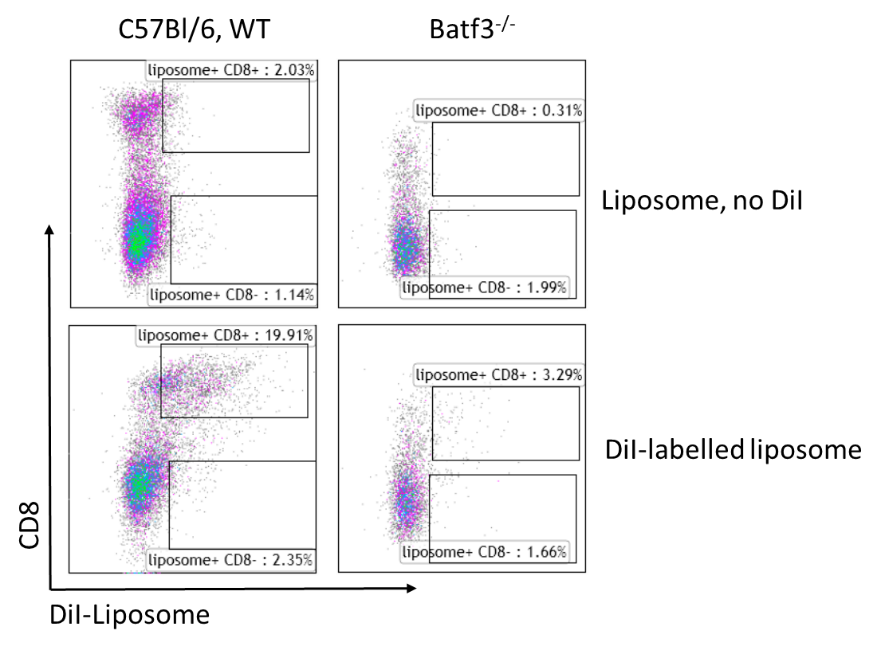


C. Gating strategy for DC and lymphocyte cell surface characteristics (Figures 2-4).

singlets

singlets

pp 81.2%

Live 99.5%

T cells: 17.06%

B cells: 57.38%

cDC1 and cDC2

B and T cells


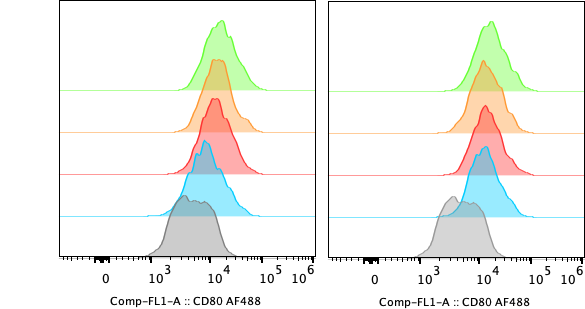


Injection vol. μL

0

25

50

100

200

Non-targeting-MSA-1 liposome

WH-MSA-1 liposome

cDC1

cDC1

CD80

D. Gating strategy for T cell polyfunctionality assay (Figures 6, 7).


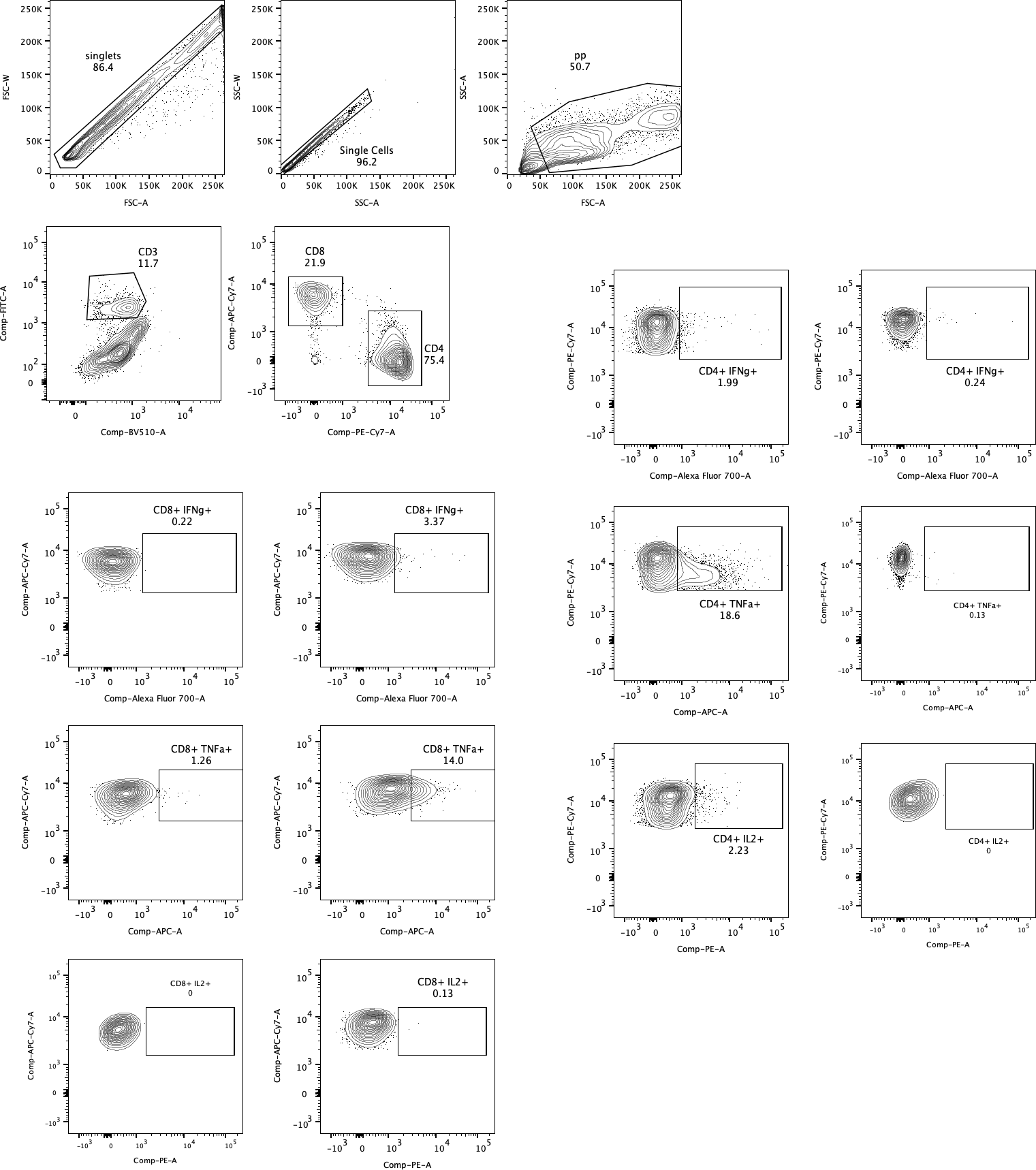


FMO CD8 IFNγ

FMO CD8 TNFα

FMO CD8 IL2

FMO CD4 IFNγ

FMO CD4 TNFα

FMO CD4 IL2

# Supplementary Figure 2. Intravenous administration of Clec9A-functionalized STING or RIG-I liposomes activates IFN-dependent pathways systemically and in the tumor

WH-liposomes encapsulating MSA-1 or RIG-I were administered to B16F10 tumor-bearing mice in the volumes shown. RNA was prepared from organs as shown and from DCs transfected with either a negative control RNA (negative control) or with a RIG-I agonist (positive control) then analyzed using a custom Nanostring nCounter IFNα/β Codeset per manufacturer’s instructions. Expression level Z-scores were normalized separately within each organ and plotted on a heat map.
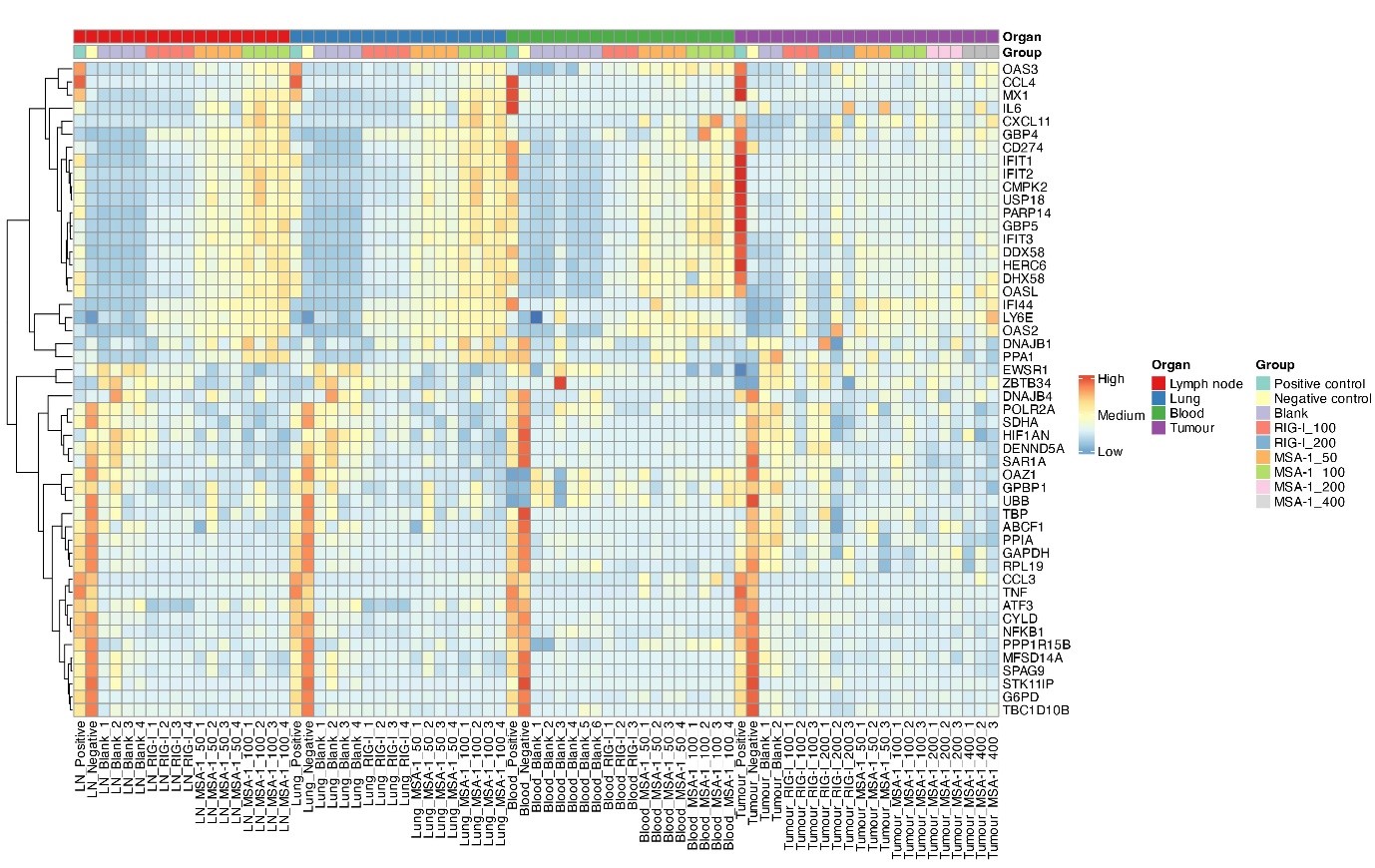

Supplement: Supplementary file 1 — Supplementary table 1 Supplementary table 2 Supplementary table 3 Supplementary table 4 Supplementary figure 1 Supplementary figure 2 [file CTI2-15-e70110-s001.docx]
